# Recovery following discharge from intensive care: What do patients think is helpful and what services are missing?

**DOI:** 10.1371/journal.pone.0297012

**Published:** 2024-03-18

**Authors:** Brenda O’Neill, Natasha Green, Bronagh Blackwood, Danny McAuley, Fidelma Moran, Niamh MacCormac, Paul Johnston, James J. McNamee, Claire Shevlin, Judy Bradley

**Affiliations:** 1 Centre for Health and Rehabilitation Technologies, INHR, Ulster University, Belfast, United Kingdom; 2 School of Medicine, Dentistry and Biomedical Sciences, Wellcome-Wolfson Institute of Experimental Medicine, Belfast, United Kingdom; 3 Antrim Hospital, NHSCT, Antrim, United Kingdom; 4 Royal Victoria Hospital, BHSCT, Belfast, United Kingdom; 5 Craigavon Area Hospital, SHSCT, Craigavon, United Kingdom; University of Chichester - Bishop Otter Campus: University of Chichester, UNITED KINGDOM

## Abstract

**Background:**

Recovery following critical illness is complex due to the many challenges patients face which influence their long-term outcomes. We explored patients’ views about facilitators of recovery after critical illness which could be used to inform the components and timing of specific rehabilitation interventions.

**Aims:**

To explore the views of patients after discharge from an intensive care unit (ICU) about their recovery and factors that facilitated recovery, and to determine additional services that patients felt were missing during their recovery.

**Methods:**

Qualitative study involving individual face-to-face semi-structured interviews at six months (n = 11) and twelve months (n = 10). Written, informed consent was obtained. [Ethics approval 17/NI/0115]. Interviews were audiotaped, transcribed and analysed using template analysis.

**Findings:**

Template analysis revealed four core themes: (1) Physical activity and function; (2) Recovery of cognitive and emotional function; (3) Facilitators to recovery; and (4) Gaps in healthcare services.

**Conclusion:**

Patient reported facilitators to recovery include support and guidance from others and self-motivation and goal setting, equipment for mobility and use of technology. Barriers include a lack of follow up services, exercise rehabilitation, peer support and personal feedback. Patients perceived that access to specific healthcare services was fragmented and where services were unavailable this contributed to slower or poorer quality of recovery. ICU patient recover could be facilitated by a comprehensive rehabilitation intervention that includes patient-directed strategies and health care services.

## Introduction

The complex nature of recovery following critical illness underpins the need for an in-depth understanding of the challenges which patients face. This is particularly important as persistent physical, psychological and cognitive deficits are highly prevalent [1–4] and contribute to readmission, mortality, and increased healthcare costs [5–7], often referred to as Post Intensive Care Syndrome (PICS) in literature [8]. To advance outcomes after critical illness, it may be helpful to explore patients’ views about what factors may have positively contributed to their recovery and whether there were any gaps in services. This could inform the need for, and timing of rehabilitation interventions that could enhance recovery.

Qualitative research is a useful methodology to collect and analyse in-depth information from patients [9]. Recently, there has been an increase in qualitative research on experiences and challenges patients face following critical illness [10, 11]. This has primarily focused on exploring patient’s perceptions of recovery in the early stages (up to six months) after intensive care unit (ICU) discharge and often at one timepoint [12, 13]. Few studies have explored patient views about recovery over a longer period of time, yet a recent review has highlighted the importance of identifying support needs following ICU discharge, as these can change throughout the recovery trajectory [14] and problems associated with PICS can last for months or even years [8, 15]. Whilst the critical care community is increasingly aware of the long-term problems associated with an ICU admission, there remains a general lack of services to address these problems [24].

Identifying, patients’ perceptions about facilitators of recovery and gaps in service delivery may be important to inform future service development for the post ICU population. A lack of explanation of disease events and of expectations about progress during critical illness and recovery, as well as poor or absent transition of care between the primary hospital and discharge destination, have been reported [13].

The aim of this study is to explore the views of patients after discharge from ICU about their recovery at six and twelve months and factors that facilitated recovery, and to determine additional services that patients felt were missing during their recovery. It is anticipated that the results of the study will inform future patient centred strategies that can support recovery following discharge from ICU.

## Methods

Face-to-face semi-structured interviews were conducted at six and twelve months with patients attending an outpatient assessment clinic as part of an observational study which included measurements taken at six weeks, six months and twelve months between November 2018 and December 2019.

Patients aged ≥ 18 years old, with an ICU admission including mechanical ventilation ≥ 48 hours, who were discharged home (self-care/carer) within 12 weeks were included in the study. Exclusion criteria were patients with a clear negative disease trajectory, existing specialist care pathway (e.g., stroke unit), declined consent or unable to give consent.

All patients satisfying the eligibility criteria (n = 15) were provided with written information about the study. The study visits took place at one of five participating hospitals in Northern Ireland. Demographics (Table 1) and objective measurements (UK FIM + FAM Motor and Cognitive scores, step counts, MSWT distance and handgrip strength score) were collected from participants during their follow up appointments. An interview guide (S1 File) was developed in line with the aims of this study, to explore the participants’ perceptions of their recovery and what helped facilitate recovery at six and twelve months. Where helpful, we used the objective measurements to guide part of the interview discussion to identify if some measurements (such as items within the FIM + FAM) had changed and to explore what contributed to this change. Following training, semi-structured interviews were conducted by NG, JB and BON. Interviews were audiotaped and transcribed verbatim by an independent transcriber. Written, informed consent was obtained from each participant and again verbally before each interview. Ethical approval was obtained from the Northern Ireland Research Ethics Committees (REC Reference: 17/NI/0115).

**Table 1:** Characteristics of participants completed interviews at 6 months (N = 11).

**Age (years)**	53.2 (15.2)
**Gender (Male/Female)**	6 [63.6] / 5 [36.3]
**ICU Category**	
Respiratory System	3 [27.2]
Sepsis	2 [18.1]
Central Nervous System	1 [9.0]
Cardiovascular System	1 [9.0]
GIT/Hepatology	2 [18.1]
Renal	1 [9.0]
Other (Diabetic Ketoacidosis)	1 [9.0]
Clinical Frailty Scale (1–9, 1 best to 9 worst)	4.0 (1.3)
APACHE score (range)	16.5 (27)
ICU LOS (days)	9.7 (3.9)
Mechanical ventilation duration (hours)	75.2 (66.2)

Results are mean (SD) or frequency [%]

This research is reported in accordance with the consolidated criteria for reporting qualitative studies (COREQ) guidelines [16] (S1 Checklist).

### Data analysis

Transcripts were analysed using template analysis (NG, JB, BON, NM) as described by King et al. [17] (S2 File). A list of codes were produced (template) representing themes identified in the textual script (S3 File). Some of these codes were defined before analysis but were often modified and expanded as the researcher read and interpreted the text as per King [18]. When all transcripts had been analysed, the coding was checked by independent reading of each patient’s transcript. In addition, the content was checked for accuracy and to ensure that all themes from the interviews were captured (NG, JB, BON, NM).

## Results

Patient characteristics of participants who completed interviews at six months are shown in Table 1. (Individual participant demographics can be found in S4 File). This was a heterogenous population, with a mean age of 53.2 years and a clinical frailty score of four indicating a vulnerable population who were in the early stage of hospital discharge.

Four core themes were identified through analysis of the interview transcripts; 1. Recovery of physical activity and function, 2. Recovery of emotional and cognitive function, 3. Facilitators to recovery, 4. Gaps in healthcare services.

### Core theme 1: Recovery of physical activity and function

Early recovery (up to six months): Early physical recovery was hindered by symptoms such as pain, weakness and dizziness, affecting patients’ ability to walk, engage in hobbies, and daily activities. Other physical problems included vocal cord damage and breathlessness. Due to low energy levels, patients described prioritising daily activities, such as choosing to go to work, instead of the gym. While some patients reported feeling better physically, others reported prolonged recovery and no changes to their physical function compared to early post discharge and others hoped to be doing better with regards to their physical function.

“*The recovery took longer for me than I anticipated*. *But I was very patient*. *I’m normally patient anyway but I was very patient with myself which was a surprise for everybody because I did hope to get back to work at Easter*. *But I just wasn’t*, *well enough*” (C2-04)

Patients described various barriers relating to the recovery of physical activity following post-hospital discharge, including the weather, fear of getting sick again and a lack of access to equipment.

*“Well*, *the other things that come to mind is*, *I suppose a fear*. *A fear*. *Because in the past*, *this has happened to me in the past*, *when I was well*, *so this happening to me now that I am not well*, *I need to be very careful it’s not that I’m not well*, *but I’m not as clued in maybe as I should be*.” *(C1-07)*

Ongoing recovery (up to twelve months): Although improvement was evident between six and twelve months, this was described as an ongoing process. At twelve months some patients felt they were still recovering and that their physical health conditions were still holding them back. Problems they were still experiencing included tiredness, weakness and low energy levels. Other patients felt physically worse when reflecting on their previous visit (six months); despite this, they described doing more, such as trying to maintain physical activity and activities of daily living since their last visit. Patients stated that the recovery process took longer than expected as some hoped to be back at work sooner.

Other patients stated that they no longer required support from a carer and highlighted slight improvements, including improved balance, less reliance on equipment, and technology and health apps to help facilitate their recovery of physical activity and function. At twelve months, the use of physiotherapy services and attending pulmonary rehabilitation classes had facilitated the recovery of physical activity for some patients.

*“Still progressing*. *Sill not 100 per cent*. *But definitely*, *even in work I’m more active than I would have been*.” (*C5-03)*

### Core theme 2: Recovery of cognitive and emotional function

Early recovery (up to six months): Patients described experiencing challenges to cognitive functioning at six months, including: forgetting how to write, gaps in memory, remembering names, recognising faces, think clearly and difficulty answering questions, leaving patients exhausted and reliant on others for support. Patients also reported feelings of uncertainty with regards to regaining their normal cognitive function. In contrast there were some participants who felt improvements with memory and concentration levels had occurred since hospital discharge, as well as feeling mentally better.

*“You know*. *But that there and memory is probably the things that I’ve noticed the most*. *Concentration and memory*” (*C5-01)*

Patients also reflected on the negative emotions experienced whilst in hospital and following discharge home; they felt traumatised, as if they did not matter, had difficulties with coming to terms with reality and experienced weird dreams.

*“There were some crazy thoughts*. *You know*. *Didn’t realise how bad I was*” (*C2-02)*

Furthermore, patients described feeling depressed and mentally weaker due to having a prolonged hospital stay. Some patients tried to keep negative thoughts out of their mind as much as possible. In contrast, others described positive memories, and some felt the ICU/hospital experience was needed to help them get back on the right track.

*“I was so sick I needed something and that was the place that got me the transition of being better and being alive*, *so I think I don’t have bad memories*, *thank*, *touchwood that I don’t*. *So*, *it was positive*” (*C2-04)*

Ongoing recovery (up to twelve months): By twelve months, patients described feeling mentally better than at six months. However, they described feeling as though they had been through a battle. Patients reported experiencing fewer flashbacks and panic attacks due to self-help, putting negative thoughts to the back of their mind and being busy with work. Patients also described speed increases with spelling and memory and feeling more focused. They were less reliant on healthcare services, including occupational therapy, psychological support and well-being classes, and technology such as health apps to relieve anxiety due to sleeping better. Some patients were back to full-time work, and had returned to their pre-ICU activities.

In contrast, some participants still experienced memory and concentration issues such as forgetfulness and short-term memory loss and some felt their memory and concentration levels were steadily getting worse, and described not feeling the same (cognitively) when compared to their pre-ICU status. Thus, highlighting the need for psychological support.

*“Yeah not using it (health apps to relieve anxiety)*. *I still have it*. *I just don’t find I need it*. *As much now*. *Used to be that I would sort of take little panic attacks and my sleeping is much better than it was now*” (*C5-03)*

### Core theme 3. Facilitators to recovery

Participants described several factors that facilitated recovery which were largely consistent across time points.

Factors facilitating recovery included receiving support from family, carers, and healthcare professionals, including; dieticians, occupational therapists, physiotherapists, psychologists and general practitioners (GPs). Participants also found that attending wellbeing classes and taking part in cognitive behaviour therapy facilitated recovery. Nutritional support such as receiving nutritional drinks and taking daily vitamins and maintaining a good diet following hospital discharge were also highlighted as a facilitator to recovery. Some participants, however, were less reliant on healthcare professionals at twelve months, as they felt their needs had been addressed.

*“I‘ve got a lot of support from friends and family*, *things like that there*.” (*C1-03)*

Participants suggested having an awareness of their lowered physical activity levels and functional capabilities motivated them to do more physical activity. Subsequent gradual increases in muscle strength resulted in the ability to walk more. They described setting positive goals as well as having the motivation to increase their physical activity and return to activities they previously enjoyed, allowing them to enjoy themselves and relax more, in addition to supporting the recovery of their mental health. Engaging in sport, exercise, attending the gym and using equipment, such as the use of a stick to facilitate walking, were important to help physical recovery and doing some of these activities with friends was noted positively. As a result of exercise and physical activity patients felt more confident and stronger and having a positive attitude further helped their recovery of both physical and mental health.

*“I think just being more physically stronger*, *has helped me mentally*” (*C5-04)*

The use of modern technology and information found online facilitated recovery of physical activity and function. Participants used health and fitness applications to track daily steps and relieve stress, and used a meditation application which helped physically and mentally.

Specific suggestions to facilitate improvement of cognitive function and mental health included giving tasks more time, waiting until a lost memory came back to them, easing back into work and trying to focus on achievement. Engaging and talking with people, listening to music, having a positive attitude, reading, making notes, and forcing themselves to remember things were important too. Repetition of activity was seen as helpful as well as eating a better diet, engaging in exercise and the use of digital technology, including health apps to relieve anxiety and playing games to improve concentration. Watching quiz programmes, setting reminders on mobile phones, enrolling in online courses, as well as pushing themselves and setting goals were some additional facilitators described. Planning for activities including hospital outpatient appointments facilitated recovery by providing a reason to plan ahead.

*“Put things in my phone to remind me and yeah I suppose I do more of these things I wouldn’t have done you know reminders on my phone just to keep track of things*” (*C5-03)*

Some patients believed their improvements were due to natural recovery over time and others were unsure of what contributed to them feeling better over time. Throughout the recovery trajectory, patients described taking their time and feeling physically better, feeling more brave and independent over time; as a result, participants were less reliant on other people to do things for them.

### Core theme 4. Gaps in healthcare service to support recovery

Patients identified various gaps in healthcare services in supporting their recovery post discharge, which were consistent across both time points.

Patients cited the main gaps in post-recovery services were a lack of follow-up, the lack of signposting to healthcare services and the need to receive support and guidance with returning to activities of daily living, including hobbies and returning to work. The lack of access to help from specific healthcare professionals or services included; clinical psychology, referral to wellbeing services from GPs, assistance to access Citizens Advice and financial support.

A perceived lack of information regarding their recovery following critical illness was evident as some patients felt they did not fully understand their illness. A need for lay terminology was also expressed. They also described a lack of feedback about their recovery and not having the opportunity to talk to someone about this and have specific questions answered. They also wanted the opportunity to talk to someone that had been through a similar experience as they had.

The need for some form of exercise rehabilitation was identified by patients, as well as having a rehabilitation programme that included information to help understand physical limitations or how to adapt to physical limitations, in which patients are monitored during activities. Gaps also included a lack of available rehabilitation programmes for those not receiving government benefits and a lack of opportunities to get out and about again, including the need for help to be taken out at the weekends and to go for a drive.

There were some patients who were uncertain about what healthcare services were missing or what services could have facilitated their recovery.

*“…*. *if the hospital has some form of physical education*, *not entertainment but physical area whereby you can combine some form of rehabilitation services with people going out and doing out activities*. *I think it would be monitored (supervised) by someone*.” (*C1-07)*

*“If it was left up to most individuals*, *I don’t think they would help themselves*. *I think they need guidance*.*” “Encouragement from more people within the health system*.” (*C5-04)*

## Discussion

This patient centred study explored views about recovery after discharge from hospital after an ICU admission, and particularly focused on facilitators of recovery during the first year. The novel findings about factors that facilitated recovery, combined with the services that were missing could be used to inform a comprehensive rehabilitation intervention that includes patient-directed strategies, and access to healthcare services that patients feel are helpful to their individual recovery needs.

The demographics of the population are broadly similar to other studies assessing patients following discharge from ICU [19, 20]. The mean clinical frailty score of the participants indicated a vulnerable population who were in the early stage of hospital discharge. Patients with scores of four to five (vulnerable/mildly frail) have been reported in other studies [21] and these scores have been associated with increased risk for ICU mortality [22]. Thus, the participants included in this study are representative of ICU populations and the study findings can be used to support patients discharged home from ICU.

This data included patients views at six and twelve months and although it is evident that some post-ICU patients have experienced some recovery over time, for others problems relating to physical activity and function, and emotional and cognitive function can still persist. Therefore, supportive strategies and follow up services may need to be accessible for at least twelve months after ICU discharge.

Patients described a diverse range of physical, emotional and cognitive problems. Recovery appeared to be individual, variable, unpredictable and complex and access to specific health care services appeared fragmented. In this study some patients independently implemented strategies to help their recovery, but it was clear that not all patients were able to do this themselves. Where healthcare services were not available this was described as contributing to slower or poorer quality of recovery. Service gaps perceived by patients included follow-up from healthcare professionals such as clinical psychology, information on accessing services, access to exercise rehabilitation, support from others that had been through a similar experience and personal feedback. Additional resources will usually be needed to facilitate the provision of these services. In the UK access to follow up services has increased across the last decade although challenges remain such as lack of dedicated staff, lack of funding, varied content, timing of service provision and omission of specific health professionals [23]. While the present study has helped to identify facilitators of recovery and services that are needed, it remains unclear how and when to best provide follow up services, rehabilitation, information and support [14, 23, 24].

Some patients were able to facilitate their own recovery by integrating the use of equipment, such as walking aids to increase their mobility, and technology to listen to music or access health apps to manage stress and anxiety. Others were less confident to do this and relied on guidance from family or health professionals e.g. occupational therapy, clinical psychology, GP. To fill these gaps for support and services, individual patient assessment could personalise recovery strategies according to patient needs [19, 25]. Identifying self-referral opportunities could be sufficient for others, especially if they are highly motivated [26]. Self-motivation and goal setting were helpful for some patients; therefore, interventions are recommended where clinical teams support and improve effective management and self-management by communicating with patients, clarifying the illness, supporting treatments, and helping with action plans have potential to be successful. The positive benefits associated with goal setting support the findings of Leventhal, Philips and Burns [27] and Leventhal’s Common-Sense Model of Self-regulation [28].

Patients expressed the need to talk to someone about their recovery or someone that has been through a similar experience. Chaboyer and Grace [29] described how survivors of critical illness gained comfort from identifying with others’ experiences, and this helped normalise their own experience. Other post ICU patients have highlighted the need for information to enable them to understand their ICU admission and their current health status during and post-hospital discharge [30]. Providing the opportunity for post-ICU patients to ask questions and gain knowledge of their intensive care stay does provide some security for the post ICU population [31]. Patient support groups, follow up clinics, and provision of ICU diaries could be embedded to provide opportunity to meet others, and provide this information.

Overall, the study findings provide a range of strategies that could be used to fill the perceived gaps that patients identified and to support patient recovery in the future. We have organised these strategies according to support needs categories that have been identified in other research studies in this area (Table 2) [14, 19, 30, 32]. This includes ways to support informational needs, such as signposting to other services; support for emotional needs, e.g. by provision of a support group to meet with others who have been through a similar experience; methods to support instrumental needs, e.g. provision of equipment; use of technology; strategies to support appraisal needs, e.g. provision of a follow-up service, setting goals; and support for spiritual needs, e.g. understanding beliefs, positive attitude. This information could help other researchers and clinicians to devise services to implement these strategies. Several methods could be considered including patient led strategies, services directed by healthcare professionals [24], exercise and rehabilitation classes [33], patient manuals [34], digital health [35], ICU diaries [36, 37], visiting ICU [34], support groups [38] or a combination of these.

**Table 2:** Strategies that could support recovery aligned to patient support needs.

Strategies that could support recovery and rehabilitation (Examples below)	
**Informational support strategies**	• How to access services e.g. healthcare services, citizens advice, finance
**Emotional support strategies**	• Peer support from others that had been through a similar experience • Support from carers and family • Support from healthcare professionals • Technology e.g. apps for relaxation, or anxiety • Setting goals • Self motivation
**Instrumental support strategies**	• Equipment to support mobility/exercise • Exercise, physical activity and rehabilitation • Nutritional support • Technology e.g. fitness apps, step tracker, online information • Cognitive and well-being strategies e.g. giving tasks more time, setting reminders, listening to music, making notes, games and quizzes for concentration • Easing back into things e.g. work, hobbies, community activities • Setting goals
**Appraisal support strategies**	Provision of a follow-up service • Provision of individual feedback • Support from healthcare professionals • Time • Setting goals
**Spiritual support strategies**	• Self motivation • Positive attitude • Beliefs

In summary, implementation of facilitatory strategies and missing services that were highlighted by patients could contribute to improvements in patient outcomes. The small sample may limit application of the results to geographic areas where patients experienced other opportunities to help recovery. However, other qualitative studies with small sample sizes have similarly provided rich and deep insights into patient experiences of recovery [39]. The strengths of this study include the exploration of both medium and longer-term perspectives of recovery (six months and twelve-months) following post-hospital discharge along with robust data cross-checking and analysis process helped to enhance the trustworthiness of the findings [17].

## Conclusion

Patients described physical, emotional and cognitive problems up to twelve months after ICU discharge. Patient reported facilitators to recovery include support and guidance from others and self-motivation and goal setting, equipment for mobility, and use of technology. Barriers include a lack of follow up services, exercise rehabilitation, peer support and personal feedback. In this study, access to specific health care services was fragmented and where healthcare services were not available this was described as contributing to slower or poorer quality of recovery. ICU patient recovery could be facilitated by a comprehensive rehabilitation intervention that includes patient-directed strategies and health care services. Rehabilitation components should be individualised and accessible for at least twelve months following ICU discharge.

## Supporting information

S1 Checklist COREQ checklist. (DOCX)

S1 File Interview guide. (DOCX)

S2 File Step by step process of template analysis. (DOCX)

S3 File Template for data analysis. (DOCX)

S4 File Participant demographics. (DOCX)
