# Supplementary material for: Recovery following discharge from intensive care: What do patients think is helpful and what services are missing?
Source: PLoS One. 2024 Mar 18;19(3):e0297012. doi: 10.1371/journal.pone.0297012 (PMC10947670; doi:10.1371/journal.pone.0297012)
Supplement: S1 Checklist — (DOCX) [file pone.0297012.s001.docx]

**S2** **– Consolidated criteria for reporting qualitative studies (COREQ): 32-item checklist [Tong et al. 2007].**

| **Topic** | **Item No.** | **Guide Questions/Description** | **Reported on**  **Page No.** |
| --- | --- | --- | --- |
| **Domain 1: Research team and reflexivity** | | | |
| *Personal characteristics* | | | |
| Interviewer/facilitator | 1 | Which author/s conducted the interview or focus group? | Page 5 - NG, BON, JB |
| Credentials | 2 | What were the researcher’s credentials? E.g. PhD, MD | NG - BSc, MSc, PhD; BON – BSc, PhD; JB - BSc, PhD |
| Occupation | 3 | What was their occupation at the time of the study? | PhD student, Senior Lecturer in Physiotherapy, Director of Northern Ireland Clinical Research Facility |
| Gender | 4 | Was the researcher male or female? | All three researchers were female |
| Experience and training | 5 | What experience or training did the researcher have? | NG - PhD Training, attended ‘Introduction to Qualitative Research Methods' course at Queens University Belfast. BON – University professor with significant qualitative research experience. JB – Professor and director of Northern Ireland Clinical Research Facility with significant qualitative research experience. NM – BSc and MSc training in qualitative research methods. |
| *Relationship with participants* | | | |
| Relationship established | 6 | Was a relationship established prior to study commencement? | No |
| Participant knowledge of the interviewer | 7 | What did the participants know about the researcher? E.g. personal goals, reasons for doing the research | The participant knew the reasons for doing research (to explore patient views about facilitators to recovery and whether the results could inform the development of future services for individuals post ICU). |
| Interviewer characteristics | 8 | What characteristics were reported about the inter viewer/facilitator? e.g. Bias, assumptions, reasons and interests in the research topic | The team were interested in exploring patient views about facilitators to recovery and whether the results could inform the development of future services for individuals post ICU. If an independent person had conducted the interviews and analysed the data this may have reduced any potential unintended bias from the research team. However, the interviewer was trained to deliver the interviews, used a schedule of topics to guide the conversation and was observed by a 2nd member of the team on several occasions to ensure fidelity of the delivery of the interviews. |
| **Domain 2: Study design** | | | |
| *Theoretical framework* | | | |
| Methodological orientation and Theory | 9 | What methodological orientation was stated to underpin the study? e.g. grounded theory, discourse analysis, ethnography, phenomenology, content analysis | Pg 5: Template Analysis |
| *Participant selection* | | | |
| Sampling | 10 | How were participants selected? e.g. purposive, convenience, consecutive, snowball | Pg 4: Participants were recruited through an outpatient assessment clinic |
| Method of approach | 11 | How were participants approached? e.g. face-to-face, telephone, mail, email | Pg 4: Face-to-face |
| Sample size | 12 | How many participants were in the study? | Pg 4: 15 Participants |
| Non-participation | 13 | How many people refused to participate or dropped out? Reasons? | Pg 4: 4 people dropped out at 6 months and 5 people dropped out at 12 months. |
| *Setting* | | | |
| Setting of data collection | 14 | Where was the data collected? e.g. home, clinic, workplace | Pg 4: outpatient assessment clinic |
| Presence of non participants | 15 | Was anyone else present besides the participants and researchers? | No |
| Description of sample | 16 | What are the important characteristics of the sample? e.g. demographic  data, date | Pg 4: Post ICU patients, 48 hrs mechanical ventilation, aged 18+ |
| *Data collection* | | | |
| Interview guide | 17 | Were questions, prompts, guides provided by the authors? Was it pilot tested? | Yes, as per S1 – Interview guide. Yes, the interview guide was pilot tested. |
| Repeat interviews | 18 | Were repeat interviews carried out? If yes, how many? | No, repeat interviews were not carried out. |
| Audio/visual recording | 19 | Did the research use audio or visual recording to collect the data? | Pg 5: The interviews were audio recorded. |
| Field notes | 20 | Were field notes made during and/or after the inter view or focus group? | No |
| Duration | 21 | What was the duration of the interviews or focus group? | Mean duration: 24.1 +/- 11.8mins |
| Data saturation | 22 | Was data saturation discussed? | No, however page 5 refers to data analysis process – the researchers checked for accuracy and content validity to ensure that all themes from the interviews were captured. |
| Transcripts returned | 23 | 23 Were transcripts returned to participants for comment and/or correction? | No |
| **Domain 3: analysis and findings** | | | |
| *Data analysis* | | | |
| Number of data coders | 24 | How many data coders coded the data? | Pg 5: 4 coders; NG, BON, JB, NM |
| Description of the coding tree | 25 | Did authors provide a description of the coding tree? | Yes, as per S5. |
| Derivation of themes | 26 | Were themes identified in advance or derived from the data? | Yes, themes were identified in advance, based on review of the first two transcripts |
| Software | 27 | What software, if applicable, was used to manage the data? | NA |
| Participant checking | 28 | Did participants provide feedback on the findings? | No |
| *Reporting* | | | |
| Quotations presented | 29 | Were participant quotations presented to illustrate the themes/findings? Was each quotation identified? e.g. participant number | Yes. Results section, pg. 6-13 inclusive. |
| Data and findings consistent | 30 | Was there consistency between the data presented and the findings? | Yes |
| Clarity of major themes | 31 | Were major themes clearly presented in the findings? | Yes. Results section, pg. 5-13 inclusive. |
| Clarity of minor themes | 32 | Is there a description of diverse cases or discussion of minor themes? | Yes. Results section, pg. 5-13 inclusive. |
