# Supplementary material for: Recovery following discharge from intensive care: What do patients think is helpful and what services are missing?
Source: PLoS One. 2024 Mar 18;19(3):e0297012. doi: 10.1371/journal.pone.0297012 (PMC10947670; doi:10.1371/journal.pone.0297012)
Supplement: S1 File — (DOCX) [file pone.0297012.s002.docx]

**S1. Interview Guide - Summary of topics explored in semi-structured interviews**

| **Introduction**  “We are interested in how you’ve been getting on since being in the intensive care unit. If you are happy to continue with a short discussion, I would just like to check if it is ok that we audio record this part of the visit?”  **Examples of probes:**  **-** What about ....? **-** Can you explain that a bit more?  **-** You mentioned ..., tell me more about that. **-** Why did you decide ... ?  **-** In what way? **-** Can you give me some examples ...?  **-** Can you explain to me why you think that? **-** How did/do you feel about that?  **6 month interview**   - How have you been managing since being in the intensive care unit? - Since going home what do you think influenced ….. (explore activities identified by the patient)? - Reflect on results from objective measures (UK FIM + FAM Motor and Cognitive scores, step counts, MSWT distance and handgrip strength score) and ask participant about any changes or lack of and what they think has contributed to this.   **Prompts as relevant**   - Are there any things/services that have helped you be able to do these activities [examples relating to physical function, getting dressed, bathing, grooming, exercise, walking] since being in the intensive care unit? - Are there any things/missing services that have stopped you from getting better at these activities [physical examples: getting dressed, bathing, grooming, exercise, walking] since being in the intensive care unit? - Are there any things/services that have helped you to do these activities [examples related to memory, concentration, emotional status] since being in the intensive care unit? - Are there any things/missing services have stopped you improving with…..[ examples related to memory, concentration, emotional status] since being in the intensive care unit? |
| --- |
| **12 month interview**   - During the previous interview, you told us you were having difficulty with **[insert physical/functional/walking/memory/concentration/emotional examples from the previous interview notes (6mths)]** and you mentioned you were doing ….**[insert examples the patient had been doing from the previous interview notes (6mths)]** to help you with physical function/activity/walking/memory/concentration/emotional recovery. - **Prompts 1A:** Are you still doing these activities/things **[insert examples]** to help you? Have you been doing anything else to help you? - **Prompts 1B:** Do you think there are any services or things missing that maybe that would help you to improve your **[insert examples]** at all? - Repeat 1A and 1B replacing examples to include relevant physical, functional, emotional and/or cognitive examples |
