# Supplementary material for: Recovery following discharge from intensive care: What do patients think is helpful and what services are missing?
Source: PLoS One. 2024 Mar 18;19(3):e0297012. doi: 10.1371/journal.pone.0297012 (PMC10947670; doi:10.1371/journal.pone.0297012)
Supplement: S2 File — (DOCX) [file pone.0297012.s003.docx]

S3 – Step by Step Process of Template Analysis [King, 2004] and the application to this study

Step 1: Familiarise yourself with the raw data to be analysed

First two completed 6 month transcripts were reviewed by NG, JB and BO’N

Step 2: Carry out preliminary coding of the data – start by highlighting anything in the textual data which strikes you as relevant and potentially contributing to understanding of the research question.

NG, JB and BO’N identified and agreed on codes for organising and categorising the data

Step 3: Once you have identified themes or codes in your textual data, an initial coding template can be defined.

An initial template for coding the data was formulated

Analysis of transcripts using the initial template (NG) and quality checks (JB, BO’N)

Step 4: The initial template can now be applied to further data and modified as necessary.

Initial template amended by NG; 3 additional themes were added

Step 5: Try out successive versions of the template, modifying and trying again can continue for as long as necessary to allow a rich and comprehensive representation of your interpretation of the data.

The final template for coding the data was formulated and applied to the full dataset

Step 6: Once a ‘final’ template has been defined, this is then applied to the full data set.
