# Supplementary figures and images for: Recovery following discharge from intensive care: What do patients think is helpful and what services are missing?
Source: PLoS One. 2024 Mar 18;19(3):e0297012. doi: 10.1371/journal.pone.0297012 (PMC10947670; doi:10.1371/journal.pone.0297012)

**
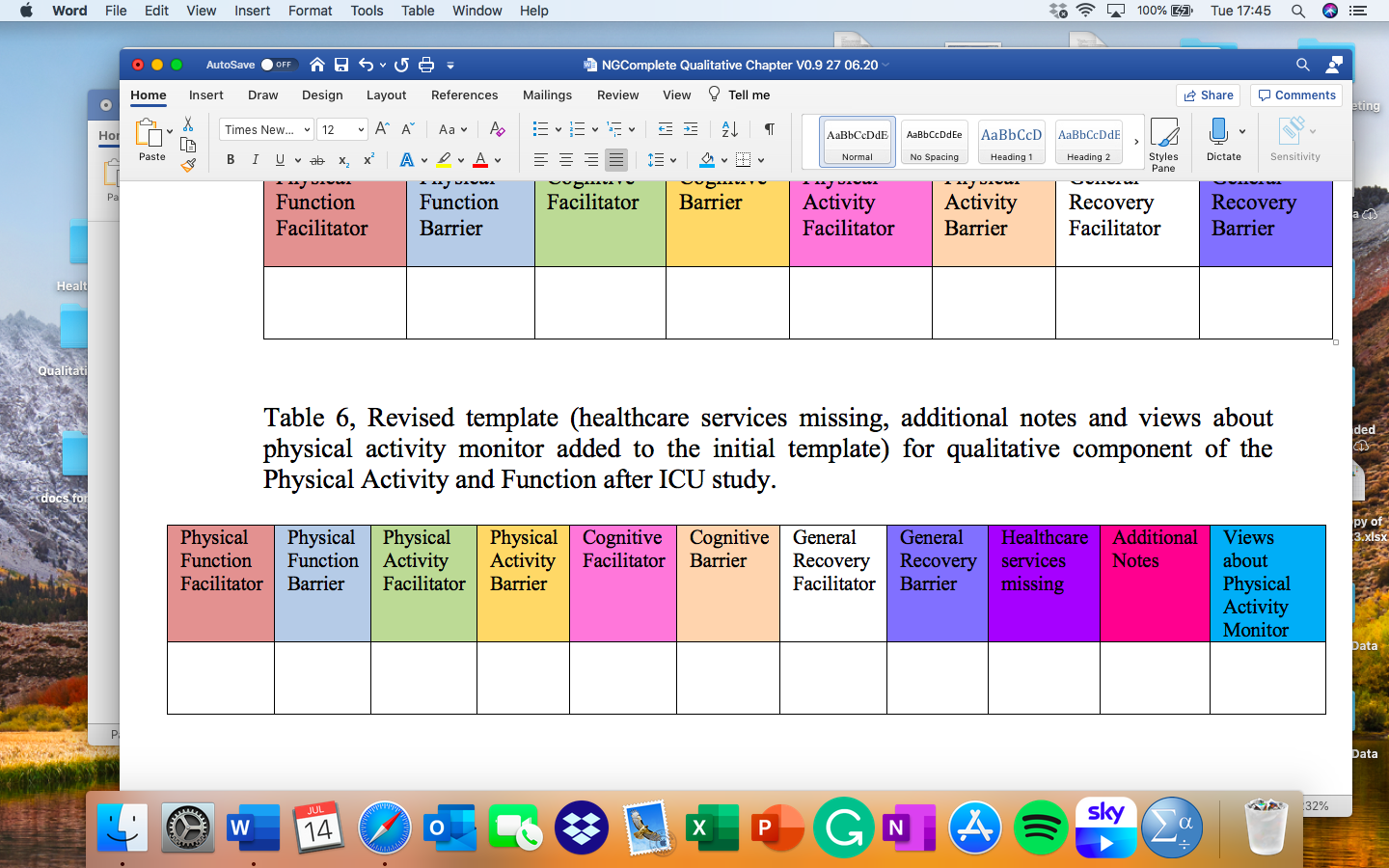
S5 - Template For Qualitative Data Analysis**

Supplement: S3 File — (DOCX) [file pone.0297012.s004.docx]
