# Supplementary material for: Recovery following discharge from intensive care: What do patients think is helpful and what services are missing?
Source: PLoS One. 2024 Mar 18;19(3):e0297012. doi: 10.1371/journal.pone.0297012 (PMC10947670; doi:10.1371/journal.pone.0297012)
Supplement: S4 File — (DOCX) [file pone.0297012.s005.docx]

| Participant ID | Gender | Age | ICU admission diagnosis | APACHE | ICU LOS (days) | Hospital LOS (days) | MV duration (hours) |
| --- | --- | --- | --- | --- | --- | --- | --- |
| C103 | Male | 34 | GIT/Hepatology | 0 | 2 | 26 | 68 |
| C107 | Female | 54 | Diabetic ketoacidosis | 25 | 12 | 28 | 164 |
| C108 | Female | 74 | Cardiovascular System | 25 | 9 | 35 | 170.5 |
| C201 | Female | 75 | Anaphylaxis & CAP | 15 | 9 | 6 | 15 |
| C202 | Male | 63 | Traumatic small bowel perforation | 27 | 11 | 66 | 27 |
| C203 | Male | 27 | Epilepsy – status epiplepticus | 10 | 9 | 12 | 10 |
| C204 | Female | 54 | Bi-lateral pneumonia | 17 | 9 | 12 | 17 |
| C501 | Male | 49 | Septic shock secondary to perforated sigmoid colon | 14 | 14 | 34 | 193 |
| C503 | Female | 40 | Abdominal sepsis | 8 | 4 | 14 | 48 |
| C504 | Male | 54 | Hypoxic post-op elective laprosopic pyloplasty – developed fluA | 25 | 12 | 28 | 23 |
| C303 | Male | 62 | Infective exacerbation COPD | 16 | 16 | 26 | 91.5 |
